# Epidermal growth factor receptor and epididymis invasion as prognostic biomarkers in clinical stage I testicular germ cell tumours

**DOI:** 10.1186/s12967-017-1162-3

**Published:** 2017-03-20

**Authors:** Miguel F. Sanmamed, E. Esteban, E. Uriol, R. Zarate, M. Capelan, C. Muriel, G. Crespo, J. P. Berros, P. Pardo-Coto, Q. Perez, C. Alvarez-Fernández, P. Jiménez Fonseca, M. Luque, A. Astudillo

**Affiliations:** 1 Department of Immunobiology, School of Medicine, Yale University, 300 George Street, Suite 203A, New Haven, CT 06511 USA; 2 Department of Medical Oncology, Hospital Universitario Central de Asturias, Oviedo, Spain; 3 Clinical Genetics Unit, Clínica Universidad de Navarra, Pamplona, Spain; 4 Breast Unit, Royal Marsden NHS Foundation Trust, London, UK; 5 Department of Medical Oncology, Hospital Regional Universitario Carlos Haya, Málaga, Spain; 6 Department of Medical Oncology, Hospital Universitario de Burgos, Burgos, Spain; 7 Department of Medical Oncology, Centro Médico de Asturias, Oviedo, Spain; 8 Department of Pathology, Hospital Universitario Central de Asturias, Oviedo, Spain

**Keywords:** Testicular germ cell tumour, EGFR, Epididymis invasion, hMLH-1, hMSH-2, MSI

## Abstract

**Background:**

Inguinal orchiectomy is curative in 70–80% of clinical stage I testicular germ cell tumours (CS I TGCT). The identification of patients who are at low risk of relapse is critical to avoid unnecessary treatment. The aim of this study is to explore EGFR, hMLH-1/hMSH-2 and microsatellite instability (MSI) as potential prognostic factors of recurrence in CS I TGCT.

**Methods:**

Fifty-six CS I TGCT patients who underwent inguinal orchiectomy were included in this study. We analysed the relationship between clinicopathological and molecular factors with survival. Analysis of hMLH1, hMSH2 and EGFR expression was carried out by immunohistochemistry. Methylation status of the hMLH1 promoter was determined by pyrosequencing analysis in selected cases. EGFR exons 19, 20, 21 were analysed by PCR labeled-fragments and MSI status was determined using standard Multiplex MSI assays.

**Results:**

Classical pathological factors such as lymphovascular invasion, high percentage of embryonal carcinoma, rete testis invasion or tumour size ≥4 cm showed a significant relationship with a higher risk of relapse. Additionally, it was found that an epididymis invasion proved to be a significant independent poor prognostic factor of recurrence (p = 0.001). hMLH1 or hMSH2 expression showed no significant association with risk of relapse and no MSI was found. EGFR expression was observed in 30.4% of samples and its expression was associated with higher risk of relapse (HR 3.5; 95% CI 1.3–9.8; p = 0.016). None of the cases presented EGFR kinase domain mutations.

**Conclusions:**

Epididymis invasion and EGFR expression, but not hMLH-1/hMSH-2 or MSI, could be potentially useful as new prognostic factors of recurrence for CS I TGCT.

**Electronic supplementary material:**

The online version of this article (doi:10.1186/s12967-017-1162-3) contains supplementary material, which is available to authorized users.

## Background

Testicular germ cell tumour (TGCT) is the most common solid malignant neoplasm among young men in Western countries [1]. Overall approximately two-thirds of patients have clinical stage I (CS I) disease at the time of diagnosis. In patients with CS I TGCT, radical inguinal orchiectomy is the treatment of choice and offers a cure rate of 70–80% [2, 3]. Current management after orchiectomy includes radiotherapy, retroperitoneal lymphadenectomy, adjuvant chemotherapy or surveillance with chemotherapy at relapse. All options have demonstrated to provide an overall survival (OS) of over 97% [4, 5]. However, in recent decades efforts have been made to reduce treatment-related morbidity and tailor adjuvant treatments to those patients who are at high-risk of relapse. Prognostic factors as lymphovascular invasion (LVI), percentage of embryonal carcinoma (EC), rete testis invasion and tumour size have been proposed to stratify patients into low and high-risk groups [4, 6, 7]. Nevertheless, several studies have reported the limited predictive value of these factors and only LVI is widely accepted [4, 8, 9].

New insights into the molecular biology of cancer have opened up new fields of research and new genetic and molecular prognostic factors have emerged. One attractive new area is DNA damage repair, where one of the most well known repair pathways is mismatch repair (MMR). Inactivation of MMR genes leads to genetic instability, characterized by small deletions or expansions within small repetitive sequences of DNA called microsatellites. This abnormality is known as microsatellite instability (MSI). MMR deficiency and MSI have been reported in TGCT [10–14]. Furthermore, a relationship between TGCT MSI and/or MMR deficiency and survival/resistance to cisplatin-based chemotherapy has been demonstrated in some studies [12–14].

Another promising group of molecular prognostic factors in oncology are growth factor receptors, and especially the epidermal growth factor receptor family member 1 (EGFR, also known as HER1 or ERBB1). EGFR is able to induce cancer through three different mechanisms: activating mutations in the catalytic domain, gene amplification or protein overexpression. Among the most widely studied mutations are a deletion in exon 19, an insertion in exon 20, and a missense mutation in exon 21, which increase the kinase activity of this growth factor receptor, conferring it with oncogenic properties [15]. EGFR expression is elevated in many tumours, which correlates with poor clinical outcome in some cases [16, 17].

In relation to TGCT, the role of EGFR is still not firmly established. Some studies have reported the expression of EGFR in 40–50% of TGCT [18–23]. Furthermore, the expression of EGFR ligands (EGF and TGF-α) has been reported in TGCTs EGFR^+^, suggesting a mechanism of cell autocrine stimulation [18]. In addition, the relation between EGFR expression and prognosis or resistance to cisplatin in advanced TGCT have been studied, with contradictory results [19, 21].

In the current study, we performed a histological review of surgically resected specimens from primary CS I TGCT who underwent inguinal orchiectomy. In order to determine new prognostic factors in these kinds of tumours we analysed the relationship between relapse-free survival (RFS) and molecular factors including MSI status, hMLH-1/hMSH-2 expression, EGFR catalytic domain mutations and EGFR expression.

## Methods

### Patients

Patients treated consecutively in our institution from 1976 to May 2016 were identified. The cases were eligible if they had (1) histological confirmation of TGCT and clinical stage I according to the American Joint Committee on Cancer (2010 edition) TNM classification (confirmed retrospectively); (2) radical inguinal orchiectomy; (3) negative levels of alpha-fetoprotein (AFP) or beta-human chorionic gonadotropin (B-HCG) 3 weeks after orchiectomy; (4) No neoadjuvant treatment or adjuvant treatment; (5) at least 1 year of follow-up.

Outpatient visits were monthly in the first year, every 3 months in the second year, each 6 months in the third to fifth year and one per year until the tenth year. At each visit serum AFP and B-HCG were measured and chest X-rays and physical examinations were performed. A computed tomography (CT) scan was performed every 3 months the first year, every 6 months the second year and once per year after the fifth year. Recurrent disease was defined as elevated serum tumour markers (AFP, B-HCG) or tumour growth as seen by radiographic study. The samples for the study were obtained through the Principado de Asturias biorepository and all patients signed a written consent.

### Tissue samples

Formalin-fixed paraffin-embedded tissue samples were retrospectively collected. The histopathological lesions of interest were analysed to select the area with more than 80% of malignant cells. All cases were reviewed by the same pathologist (AA) and classified according to WHO criteria. Histopathological review of the testis tumour included tumour size, presence or absence of LVI, rete testis invasion, epididymis invasion (EI) and predominant histological subtype. Three sections cut from tissue blocks of 0.1 mm each were mounted on tissue microarray (TMA) blocks. TMA block sections 3-μm thick were mounted on REAL™ Capillary Gap Microscope Slides (DAKO) in preparation for immunohistochemistry (IHC). Additional sections 0.2 mm thick were taken for genetic analysis.

### Immunochemistry studies

Immunohistochemical analysis was performed using the EGFR pharm Dx™ kit (DAKO, Glostrup, Denmark) and the EnVision™ FLEX kit (DAKO), according to the manufacturer’s instructions.

The sections were incubated with primary antibodies against hMLH1 (mouse anti-hMHL1: clone ES05 (DAKO), diluted 1:50, 30 min RT), hMSH2 (mouse anti-hMSH2: clone FE11 (Invitrogen), diluted 1:100, 20 min RT) and EGFR (mouse anti-EGFR: clone 2-18C9 (DAKO), diluted 1:100, 30 min RT).

The extent of staining was evaluated by visual examination microscopically. Nuclear staining (hMLH1 and hMSH2) was scored as “normal”, “low,” or “absent” compared with internal positive controls, according to previous scoring systems [12, 13]. In the EGFR analysis, the scoring system recommended by Tsao et al. [24] was followed and results were recorded as positive (staining of ≥10% of membrane cells) or negative (staining of <10% of membrane cells).

### Genomic DNA analysis

Following deparaffinization in xylenes and ethanol, DNA was extracted from the tissue scrapings using the QIAamp DNA Mini kit (Qiagen, Valencia, CA, USA).

All PCRs were carried out using Gene Amp^®^ Polymerase Chain Reaction (PCR) Systems 9700 (Applied Biosystems, Foster City, CA, USA). Negative controls were included in each set of amplifications.

All PCR data were subsequently analysed on an ABI PRISM^®^ 310 Genetic Analyzer using capillary electrophoresis.

### EGFR exon analysis

PCR and fragment analysis were performed as described previously [25], with slight modifications. Fragments of EGFR exons 19, 20 and 21 were amplified using the primer-pairs listed in Additional file 1: Table S1. The forward primers specific for exons 19 and 20 were labeled with 6-carboxyfluorescein (6-FAM) at the 5′-end. The expected amplicon size for exon 19 is 145 bp; a smaller size indicates the presence of a deletion. The expected amplicon size for exon 20 is 204 bp; the resulting amplicon will be larger if an insertion has occurred. To detect the p.L858R mutation in exon 21, an allele-specific oligonucleotide PCR was performed. Three primers were used to amplify the region encompassing the mutation. The reverse primer is labeled with 5′HEX. The expected amplicon size for exon 21 is 216 bp. If the L858R mutation is present, the allele-specific forward primer creates an additional amplicon of 147 bp.

Following PCR, 2 µl of product and 0.5 µl of GeneScan 500-LIZ molecular weight standard (Applied Biosystems, Madrid, Spain) were denatured in 15 µl of formamide at 95 **°**C for 10 min. Separation was achieved using a four-color laser induced fluorescence capillary electrophoresis system: ABI PRISM^®^ 310 (Applied Biosystems, Madrid, MA, Spain) with POP4 polymer. Evaluation was performed using GeneMapper v.3.7 software (Applied Biosystems).

### Microsatellite analysis

Multiplex analysis of MSI status of all tumour DNA samples analysed in this study was determined with the MSI Analysis System Version 1.2 (Promega). The MSI Analysis System consists of five nearly monomorphic mononucleotide markers (BAT-25, BAT-26, NR-21, NR-24, and MONO-27) for MSI determination and two polymorphic pentanucleotide markers (Penta C and Penta D) for each sample identification.

We interpreted MSI instability as high when present at ≥2 microsatellite loci, low when present at a single microsatellite locus and stable (MSS) when no instability at any of the loci assessed was found, as established in the Revised Bethesda Guidelines [26].

### Methylation of hMLH-1 promoter

The methylation status of hMLH-1 genes was analysed using a methylation-specific polymerase chain reaction (MSP) assay. Bisulfite modification of genomic DNA was carried out with the EZ DNA Methylation-GoldTM Kit (Zymo Research Corporation) following the manufacturer’s protocol.

The pyrosequencing reaction was performed on a PyroMark Q24 MDx Vacuum Workstation (Qiagen) using Pyro Gold Q24 Reagents (Qiagen) and Streptavidin Sepharose HP (Amersham Biosciences). Purification and subsequent processing of the biotinylated single-stranded DNA was performed according to the manufacturer’s recommendations. The pyrosequencing primers were used in a final concentration of 0.5 μmol/l. Resulting data were analysed and quantified with the PyroMark Q24 software version 2.0.6 (Qiagen). This software calculates the methylation percentage (mC/(mC + C)) for each of the 5 CpG islands present in the hMLH-1 promoter. The methylation score was given as a percentage of methylation on each locus, obtained by adding the percentages of each CpG on each locus. A total methylation of 15% was used as threshold for hMLH1 hypermethylation, as previously reported [27].

### Statistical methods

Relapse-free survival (RFS) and overall survival (OS) curves were estimated by the Kaplan–Meier method. Comparison of resulting relapse-free survival curves were performed using the log-rank test. Univariate and multivariate analysis was performed using the Cox proportional hazards regression model [28]. Probability values (P values) lower than 0.05 were considered statistically significant.

## Results

### Patients and specimens

Fifty-six patients with CS I TGCTs treated in our institution were included in this study. All underwent radical orchiectomy. Baseline patient and tumour characteristics are summarized in Table 1.

**Table 1:** Patient demographics and clinical characteristics

Variable		Surveillance group (N = 56) No (%)
Age-years		
Median		30
Standard deviation		8.5
Histology		
Seminoma		27 (48.2)
Pure EC		10 (19.6)
Pure Yolk Sac tumor		1 (1.9)
Mixed tumor		18 (32.1)
Tumor diameter		
≥4 cm		26 (46.4)
<4 cm		27 (48.2)
Unknown		3 (5.4)
Vascular and lymph vessels invasion		
Yes		16 (28.6)
No		38 (67.8)
Unknown		2 (3.6)
Presence of EC		
≥50%		22 (39.2)
<50%		33 (58.9)
Unknown		1 (1.9)
Rete testis invasion		
Yes		18 (32.1)
No		34 (60.7)
Unknown		4 (7.1)
Epididymis invasion		
Yes		8 (14.3)
No		48 (85.7)
Unknown		0 (0)
Stage		
IA		27 (48.2)
IB		11 (19.6)
IS		17 (30.3)
Unknown		1 (1.9)

*EC* embryonal carcinoma

Out of all the patients, 26.8% (15 from 56) relapsed with a median follow-up of 5.2 years (SD 4.3). All relapsed cases were rescued with platinum-based chemotherapy. Five-year estimated relapse-free survival and overall survival was 73.2 and 100% respectively.

### MMR immunostaining, hMLH1 promoter methylation and MSI analysis

**Fig. 1:**
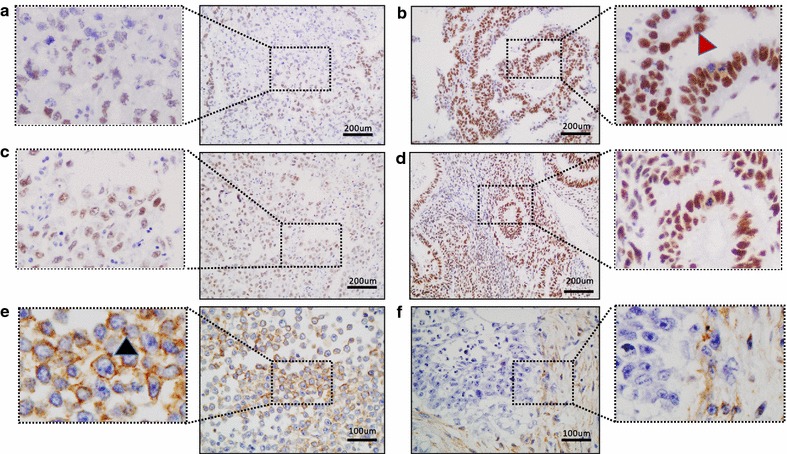
Representative immunohistochemistry images of hMLH1, hMSH-2 and EGFR protein expression. Nuclear hMLH1 staining in tumours with “low” (**a**) and “normal” (**b**) expression. Representative hMSH-2 staining in tumour with “low” (**c**) and “normal” (**d**) expression. A seminoma (**e**) and embryonal carcinoma (**f**) showing positivity for EGFR staining. A high-power view of the boxed regions is shown in the insets. Nuclear and membrane signal (*brown*) is visualized with diamino-benzidine as chromogen. The *black arrowhead* depict EGFR immunoreactivity in the membrane of a seminoma cell. The *red arrowhead* shows nuclear hMLH1 signal

Immunostaining of hMLH1 and hMSH2 showed an intense hMLH1 and hMSH2 nuclear staining in most cases, regardless of the histology (Additional file 2: Table S2). hMLH-1 expression was considered null or low in 27 (48.2%) cases and hMSH-2 in 16 (28.6%) cases (representative samples shown in Fig. 1a–d).

In order to find an epigenetic mechanism to explain a gradual expression of MMR proteins, we selected absent (n = 3), low (n = 5) and normal (n = 5) hMLH1 expression samples to study the methylation status of the hMLH1 gene promoter. No differences in average methylation percentage values between the three groups were observed, and all cases were found to be unmethylated (data not shown). Also, in the three cases where expression was absent and two cases with low levels, adjacent normal tissues were analysed. However, hypermethylation was not detected in the hMLH1 promoter of both tumoural and normal tissues (Additional file 2: Figure S1).

MSI was examined using a panel of five mononucleotide markers (BAT 25, BAT 26, NR 21, NR 24 and MONO 27). None of the samples analyzed met the criteria for MSI.

### EGFR immunostaining and genomic DNA analysis of exons 19, 20, 21

Immunostaining for EGFR was performed as described above. Seventeen (30.4%) out of 56 tumours analysed showed positive immunoreactivity to EGFR (Table 2). Different percentages of positive cases were found among different histologies: 7 (26%) of 27 seminomas, 2 (66.6%) of 3 teratomas, 8 (36.4%) of 22 EC and none of 4 yolk sac tumours studied. Moreover, different patterns of expression were evident: seminoma tumours showed membranous immunoreactivity exclusively in the parenchyma cells (Fig. 1e); embryonal tumours showed immunoreactivity restricted to the stroma instead of parenchyma (Fig. 1f); teratoma specimens showed immunoreactivity only in the epithelial components and not in mesenchymal teratomatous cells (data not shown).

**Table 2:** Summary of immunohistochemistry showing hMLH-1, hMSH-2 and EGFR expression

Expression	No. patients (N = 56)	(%)
hMLH-1		
Null	4	7.1
Low	23	41.1
Normal	29	51.8
hMSH-2		
Null	1	1.9
Low	15	26.7
Normal	40	71.4
EGFR		
Positive	17	30.4
Negative	39	69.6

None of the 56 cases assessed showed EGFR mutations in exon 19, 20 or 21.

### Assessment of risk factors and RFS

**Fig. 2:**
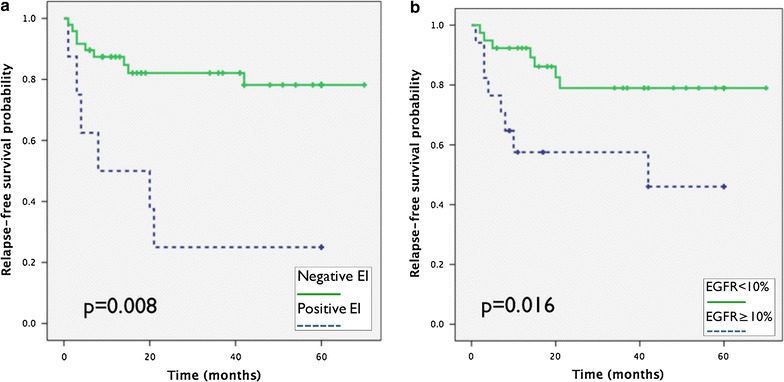
Kaplan–Meier curves representing survival of TGCT patients stratified by epididymis invasion presence (**a**) or EGFR expression (**b**). Differences between the two curves were calculated using the log-rank tests and resultant p value is shown in each graph. *EI* epididymis invasion, *NS* Non-seminoma, *ns* not significant

Univariate analysis showed that classical factors (rete testis invasion, tumour size ≥4 cm, LVI) were significantly associated with a higher risk of relapse in our series (Table 3A). Rete testis invasion or tumor size ≥4 cm in seminoma group and LVI or percentage ≥50% of EC in non seminoma group were significantly associated with higher risk of relapse (Additional file 3: Table S3). In addition, EI was associated with higher risk of relapse [Hazard Ratio (HR) 3.6; 95% confidence interval (CI) 1.4–9.3; p = 0.008] in our series (Table 3A; Fig. 2a). EI also showed a trend for higher risk of relapse when patients were stratified by histological subtypes, however it was not statistically significant (Additional file 3: Table S3).

**Table 3:** Survival analysis

Variable	Relapse-free survival		
	5 years-RFS (%)	HR (95% CI)	p value
*A. Univariate analysis*			
Histology			
NS	59.1	4.8 (1.4–16.3)	0.012
Seminoma	90.3	1	
Age			
≤30	61.5	2.5 (1–6.5)	0.056
>30	83.3	1	
LVI			
Yes	54.5	3.2 (1.3–8.2)	0.014
No	83.0	1	
Tumor size (cm)			
≥4	57.1	4.5 (1.5–13.5)	0.008
<4	87.9	1	
Rete testis invasion			
Yes	54.5	4.5 (1.5–13.3)	0.006
No	88.4	1	
Epididymis invasion			
Yes	25.0	3.6 (1.4–9.3)	0.008
No	81.3	1	
hMLH1 expression			
Null/low	66.7	2 (0.7–5.7)	0.178
Normal	79.3	1	
hMSH2 expression			
Null/low	81.3	0.66 (0.2–2.3)	0.524
Normal	70.0	1	
EGFR expression			
Positive	52.9	3.5 (1.3–9.8)	0.016
Negative	82.1	1	

Variable	Relapse-free survival	
	HR (95% CI)	p value
*B. Multivariate analysis*		
Histology		
NS		ns
Seminoma		
Age		
≤30		ns
>30		
LVI		
Yes		ns
No		
Tumor size (cm)		
≥4		ns
<4		
Rete testis invasion		
Yes		ns
No		
Epididymis invasion		
Yes	7.6 (2.4–23.7)	0.001
No	1	
EGFR expression		
Positive		ns
Negative

There was a significant association between EGFR expression and higher risk of relapse. At 5-years, in the EGFR^+^ group the RFS was 52.9% in contrast with 82.1% in the EGFR^−^ group (HR 3.5; 95% CI 1.3–9.8; p = 0.016) (Table 3A; Fig. 2b). Similar results were found when seminoma and non seminoma groups were analyzed (Additional file 1: Table S3). hMLH-1 or hMSH2 expression did not show a significant relation with risk of relapse in our series and neither by histological subtypes (Table 3A; Additional file 3: Table S3).

A multivariate analysis identified EI as an independent predictor of outcome (HR 7.6; 95% CI 2.4–23.7; p = 0.001) (Table 3B).

## Discussion

A surveillance strategy with chemotherapy at relapse has been shown to be a valid alternative to retroperitoneal lymphadenectomy, radiotherapy or adjuvant chemotherapy in patients with CS I TGCT after inguinal orchiectomy. Such a strategy has no impact on overall survival and avoids unnecessary treatment-related toxicity [2, 29]. Recent data have encouraged the use of risk-adapted management strategies [30]. Nevertheless, the absence of precise markers to predict risk of relapse in each patient after orchiectomy leads to most hospitals systematically using adjuvant treatment.

Pathological features, previously reported as useful predictors of poor outcome in TGCT have been correlated in the present study with a higher risk of relapse. Furthermore, in multivariate analysis an independent association of epididymis invasion and higher risk of relapse has been shown. Epididymis invasion has previously been reported as a significant prognostic factor by Hoskins et al. and in the Medical Research Council prospective studies [4, 31]. Nonetheless, in neither of these studies was this factor shown to have an independent value in the multivariate analyses. To our knowledge ours is the first study to show that this factor has an independent relationship with RFS.

We studied MMR expression based on previous research reporting loss of hMLH1 and hMSH2 expression as being of prognostic significance in TGCT [12–14]. Our results show absent, low and normal hMLH1 and hMSH2 expression in proportions similar to those found in other studies [11, 32, 33] (Table 2). Studies in colon cancer have found the hMLH-1 promoter to be hypermethylated in 30% of sporadic cases with loss of hMLH1 expression. Epigenetic modifications may explain different levels of protein expression and an early study has reported a 5.7% rate of hMLH1 promoter methylation in TGCTs [34]. Consequently, we decided to study hMLH-1 promoter methylation status in selected cases with absent, low and normal hMLH1 expression. However, all the studied cases were unmethylated. Therefore, another epigenetic deregulation or somatic mutation could be the cause of the lack of expression in our TGCT cases. Further investigation in this line is currently ongoing.

In contrast with Velasco et al. [13], in our experience there was no relationship between null/low hMLH1 expression and risk of relapse. The reason could be due to genetic differences between the two populations studied (caucasian vs american-caucasian) or to differences in the treatment management, as in the Velasco et al. [13] study some patients received chemotherapy prior to surgery. Well-designed prospective studies are necessary to validate hMLH1 as a useful prognostic factor. In relation with hMSH-2, our findings are in agreement with previous reports confirming the absence of relationship between its expression and risk of relapse [13].

hMLH1 and hMSH2 are responsible for genetic stability and a lack of these proteins could generate a special type of genetic instability known as MSI. Because we found null/low expression of MMR proteins in some cases, we decided to study the MSI status of our TGCT cases. Nevertheless, we did not find MSI in any of our 56 CS I TGCT cases. This result contrasts with previous studies that have reported MSI in 10–30% of TGCT. Most of these studies analysed II–IV stage TGCT cases and also some cisplatin treated patients were included [12, 14, 33]. In contrast, we selectively studied clinical stage I patients without previous chemotherapy. Different stages of TGCT could be critical in the appearance of MSI, as this process needs time to develop from MMR deficiency to the appearance of MSI. Furthermore, MSI might be secondary to chemotherapy and thus should be studied in samples from chemotherapy-naive patients.

EGFR expression was analysed in our series based on previous reports of EGFR overexpression in TGCT cases [18–23] and its value as a prognostic factor in other tumours [16, 35]. We found that almost one-third of the TGCT samples studied showed EGFR expression by IHC. Moreover, we found that 47% of EGFR^+^ patients relapsed 5-years postorchiectomy, in contrast with 18% of patients without EGFR immunoreactivity who relapsed during this period (Table 3A). Similarly, Miyai et al. [36] published the result of an EGFR study in 209 histologically distinct components from 110 TGCT cases wherein 35 (32%) showed immunoreactivity to EGFR. This study suggests that EGFR expression may be involved in the progression from “pre-invasive” lesions (IGCNU) to invasive lesions, because its expression is absent in the IGCNU tissue and is more frequent in choriocarcinoma, which represents a more aggressive phenotype of TGCTs. Also, previous studies have suggested the possible oncogenic potential of EGFR in adult TGCTs [18]. The present work describes a significant correlation between the expression of EGFR and higher risk of relapse in early stage TGCTs after orchiectomy, which might constitute further proof of the possible oncogenic potential of EGFR in TGCTs. To our knowledge ours is the first study to report a significant relationship between EGFR expression and higher risk of relapse in TGCT.

The most important limitation of this study is the sample size. Due to this limitation, we could not assess the prognosis value of EGFR expression among different TGCT histologies. This could be a possible explanation of the poor relationship between EGFR expression and the risk of relapse in multivariate analysis. Further studies with a larger representation for each TGCT subtype would be needed to evaluate the prognosis value of EGFR expression in these tumours.

## Conclusions

Mismatch repair proteins and microsatellite analysis did not correlate in this study with clinical outcome. Epididymis invasion and EGFR expression have shown a potential value as new risk factors of recurrence in CS I TGCT, which deserves to be confirmed in a prospective and larger analysis.

## Additional files

**Additional file 1: Table S1.** Characteristics of primers used in EGFR exons 19, 20 and 21 amplification.

**Additional file 2: Table S2.** EGFR, hMLH1 and hMSH2 expression in TGCT subtypes.

**Additional file 3: Table S3.** Univariate analysis Seminomas B. Univariate analysis Non Seminomas.

**Additional file 4: Figure S1.** Methylation levels result of hMLH1 promoter. Comparison between Tumour (T) and Normal adjacent tissue (N) in 5 patients. Tumors from patients 4, 27 and 43 showed absent expression levels by IHC and tumors from patients 61 and 64 showed low expression levels by IHC. Methylation percentage values are depicted for both average and individual CpG islands of hMLH-1 promoter indicated as CpG1, CpG2, CpG3, CpG4 and CpG5.

## Abbreviations

TGCT: testicular germ cell tumour
CS I: clinical stage I
EGFR: epidermal growth factor receptor
MMR: mismatch repair
MSI: microsatellite instability
IHC: immunohistochemistry
LVI: lymphovascular invasion
EC: embryonal carcinoma
EI: epididymis invasion
OS: overall survival
PFS: progression-free survival
