# Supplementary material for: Epidermal growth factor receptor and epididymis invasion as prognostic biomarkers in clinical stage I testicular germ cell tumours
Source: J Transl Med. 2017 Mar 20;15:62. doi: 10.1186/s12967-017-1162-3 (PMC5358043; doi:10.1186/s12967-017-1162-3)
Supplement: Supplementary file 1 — Additional file 1: Table S1. Characteristics of primers used in EGFR exons 19, 20 and 21 amplification. [file 12967_2017_1162_MOESM1_ESM.pdf]

Supplementary Table 1. Characteristics of primers used in EGFR exons 19, 20 and 21 amplification.

Exon	Primer	Sequence	Fluorophore	Fluorophore Final [ ]	Hibridation Temperature	Amplicon size (bp)
19	Forward	5´-GGA CTC TGG ATC CCA GAA GG- 3´	6FAM™	0,2 µM	64°C	145
	Reverse	5´-CCT GAG GTT CAG AGC CAT GG- 3´		0,4 µM		
20	Forward	5´-ATG CGA AGC CAC ACT GAC G-3´	6FAM™	0,2 µM	60°C	204
	Reverse	5´- CAC CAG TTG AGC AGG TAC TG- 3´		0,4 µM		
21	Forward	5´-GAG GAC CGT CGC TTG GTG CA-3´		0,08 µM	52°C	216 (147**)
	Forward*	5´-CAA GAT CAC AGA TTT TGG ACG-3´		0,8 µM		
	Reverse	5´- CAA TAC AGC TAG TGG GAA G-3´	HEX	0,4 µM		

*specific for mutated allele; **amplicon size when p.L858R mutation is present
