# Supplementary material for: Epidermal growth factor receptor and epididymis invasion as prognostic biomarkers in clinical stage I testicular germ cell tumours
Source: J Transl Med. 2017 Mar 20;15:62. doi: 10.1186/s12967-017-1162-3 (PMC5358043; doi:10.1186/s12967-017-1162-3)
Supplement: Supplementary file 2 — Additional file 2: Table S2. EGFR, hMLH1 and hMSH2 expression in TGCT subtypes. [file 12967_2017_1162_MOESM2_ESM.pdf]

Supplementary Table 2. EGFR, hMLH1 and hMSH2 expression in TGCT subtypes.

	hMLH1				hMSH2				EGFR		
	Nule	Low	Normal	Total	Nule	Low	Normal	Total	+	-	Total
Seminoma	**0**	**10**	**17**	27	**0**	**8**	**19**	27	**7**	**20**	27
EC	**2**	**12**	**8**	22	**1**	**6**	**15**	22	**8**	**14**	22
Teratoma	**0**	**0**	**3**	3	**0**	**1**	**2**	3	**2**	**1**	3
YST	**2**	**1**	**1**	4	**0**	**0**	**4**	4	**0**	**4**	4
Total	4	23	29	56	1	15	40	56	17	39	56
