# Supplementary material for: Epidermal growth factor receptor and epididymis invasion as prognostic biomarkers in clinical stage I testicular germ cell tumours
Source: J Transl Med. 2017 Mar 20;15:62. doi: 10.1186/s12967-017-1162-3 (PMC5358043; doi:10.1186/s12967-017-1162-3)
Supplement: Supplementary file 3 — Additional file 3: Table S3. Univariate analysis Seminomas B. Univariate analysis Non Seminomas. [file 12967_2017_1162_MOESM3_ESM.pdf]

Supplementary Table 3.

A. Univariate analysis Seminoma

Variable	Relapse-free survival		
	5 years-RFS (%)	HR (95% CI)	p-value
**Age**			
≤ 30	89	1.1 (0.1-12.4)	0.88
>30	89	1	
**LVI**			
Yes	87	1.5 (0.1-16.8)	0.7
No	90	1	
**Tumor size**			
≥4 cm	83	3.4 (1.3-8.6)	0.05
<4 cm	100	1	
**Rete testis invasion**			
Yes	67	3.5 (1.3-9.2)	0.02
No	100	1	
**Epididymus invasion**			
Yes	50	2.9 (0.9-8.9)	0.06
No	93	1	
**hMLH1 expression**			
Null/low	90	1.3 (0.1-15.5)	0.80
Normal	88	1	
**hMSH2 expression**			
Null/low	100	0.7 (0.2-2.3)	0.40
Normal	84	1	
**EGFR expression**			
Positive	71	2.7 (0.9-8.6)	0.06
Negative	95	1	

B. Univariate analysis Non Seminoma

Variable	Relapse-free survival		
	5 years-RFS (%)	HR (95% CI)	p-value
**Age**			
≤ 30	53	1.8 (0.6-5.6)	0.27
>30	71	1	
**LVI**			
Yes	36	3.5 (1.2-9.8)	0.01
No	77	1	
**Tumor size**			
≥4 cm	43	3.0 (1-9.3)	0.04
<4 cm	78	1	
**Rete testis invasion**			
Yes	50	2.3 (0.8-7.1)	0.13
No	75	1	
**Epididymus invasion**			
Yes	17	2.4 (0.8-6.6)	0.09
No	65	1	
**hMLH1 expression**			
Null/low	53	1.8 (0.5-5.8)	0.35
Normal	67	1	
**hMSH2 expression**			
Null/low	62	0.8 (0.2-2.9)	0.72
Normal	57	1	
**EGFR expression**			
Positive	40	2.7 (0.9-8.5)	0.07
Negative	68	1	
