# Supplementary figures and images for: Epidermal growth factor receptor and epididymis invasion as prognostic biomarkers in clinical stage I testicular germ cell tumours
Source: J Transl Med. 2017 Mar 20;15:62. doi: 10.1186/s12967-017-1162-3 (PMC5358043; doi:10.1186/s12967-017-1162-3)

Supplementary Figure 1.

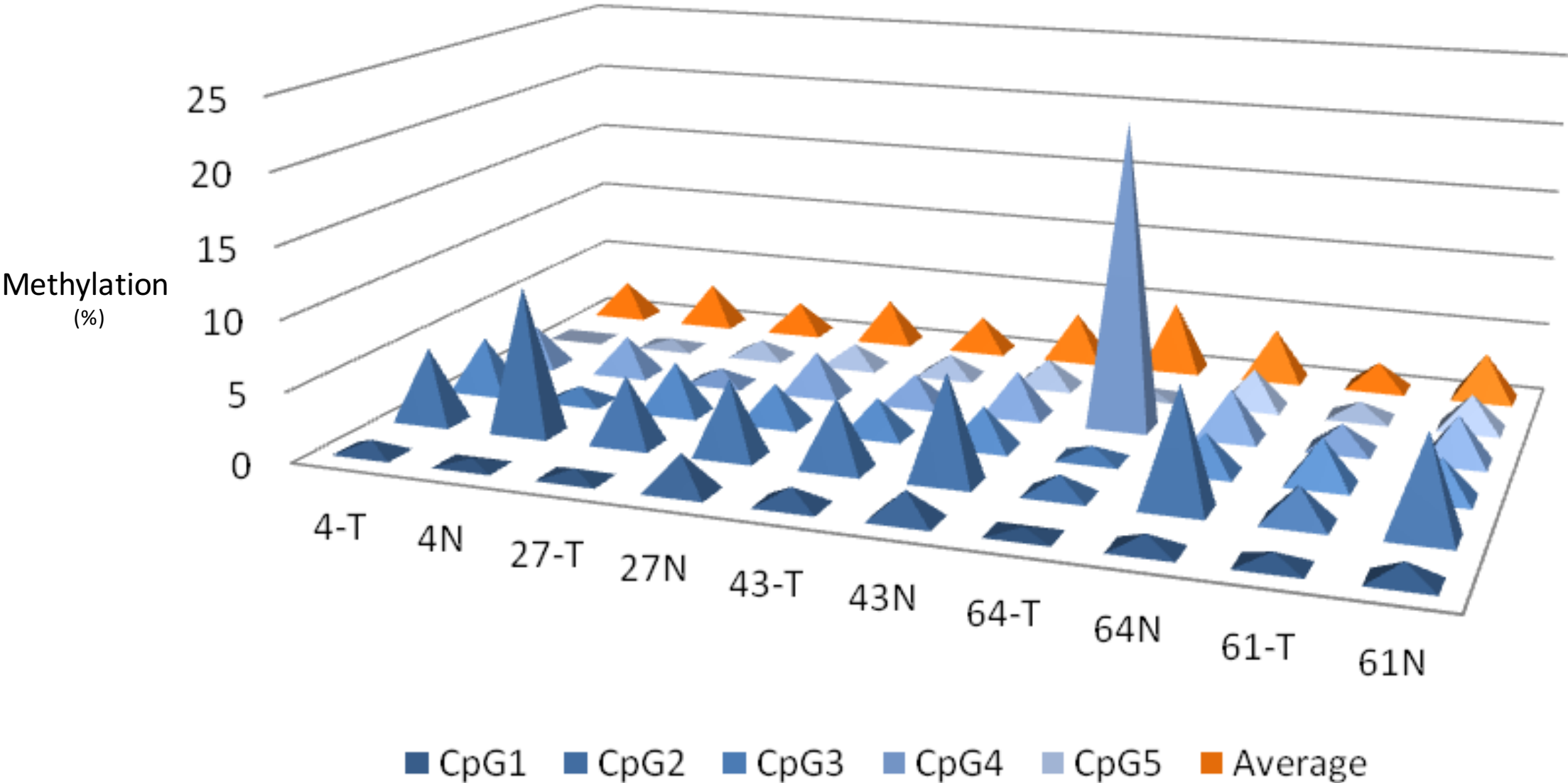

Supplement: Supplementary file 4 — Additional file 4: Figure S1. Methylation levels result of hMLH1 promoter. Comparison between Tumour (T) and Normal adjacent tissue (N) in 5 patients. Tumors from patients 4, 27 and 43 showed absent expression levels by IHC and tumors from patients 61 and 64 showed low expression levels by IHC. Methylation percentage values are depicted for both average and individual CpG islands of hMLH-1 promoter indicated as CpG1, CpG2, CpG3, CpG4 and CpG5. [file 12967_2017_1162_MOESM4_ESM.pdf]
